# Cost-efficient designs for three-arm trials with treatment delivered by health professionals: Sample sizes for a combination of nested and crossed designs

**DOI:** 10.1177/1740774517750622

**Published:** 2018-01-10

**Authors:** Mirjam Moerbeek

**Affiliations:** Department of Methodology and Statistics, Utrecht University, Utrecht, The Netherlands

**Keywords:** Crossed and nested designs, health professional, statistical power, multilevel data, intraclass correlation coefficient

## Abstract

**Background:**

This article studies the design of trials that compare three treatment conditions that are delivered by two types of health professionals. The one type of health professional delivers one treatment, and the other type delivers two treatments, hence, this design is a combination of a nested and crossed design. As each health professional treats multiple patients, the data have a nested structure. This nested structure has thus far been ignored in the design of such trials, which may result in an underestimate of the required sample size. In the design stage, the sample sizes should be determined such that a desired power is achieved for each of the three pairwise comparisons, while keeping costs or sample size at a minimum.

**Methods:**

The statistical model that relates outcome to treatment condition and explicitly takes the nested data structure into account is presented. Mathematical expressions that relate sample size to power are derived for each of the three pairwise comparisons on the basis of this model. The cost-efficient design achieves sufficient power for each pairwise comparison at lowest costs. Alternatively, one may minimize the total number of patients. The sample sizes are found numerically and an Internet application is available for this purpose. The design is also compared to a nested design in which each health professional delivers just one treatment.

**Results:**

Mathematical expressions show that this design is more efficient than the nested design. For each pairwise comparison, power increases with the number of health professionals and the number of patients per health professional. The methodology of finding a cost-efficient design is illustrated using a trial that compares treatments for social phobia. The optimal sample sizes reflect the costs for training and supervising psychologists and psychiatrists, and the patient-level costs in the three treatment conditions.

**Conclusion:**

This article provides the methodology for designing trials that compare three treatment conditions while taking the nesting of patients within health professionals into account. As such, it helps to avoid underpowered trials. To use the methodology, a priori estimates of the total outcome variances and intraclass correlation coefficients must be obtained from experts’ opinions or findings in the literature.

## Introduction

Subjects are often nested within health professionals in trials on the prevention or treatment of addiction, disease or disorder. Examples of health professionals are dentists, surgeons, psychologists and psychiatrists. As health professionals vary with respect to their skills, experience, competence and enthusiasm, it is very likely outcomes of subjects treated by the same health professional are dependent. It is therefore important that a random factor for health professional is included in the model that relates treatment condition to outcome.^1–4^

Walwyn and Roberts^5^ give an overview of developments in trials where treatment is delivered by therapists, and provide a review of different designs that can be encountered in such trials. In the *nested design*, therapists are nested within treatments, so each therapist delivers just one treatment. Such a design is often chosen to avoid the risk of contamination^6^ and may also lower costs since each therapist has to be trained to deliver only one treatment. A parallel can be drawn between a nested design and a cluster randomized trial by equating the cluster in a cluster randomized trial to a therapist in a nested design. The design and analysis of cluster randomized trials have been widely discussed in the statistical literature.^7–17^

In the *partially nested design*, there is no therapist involved in one of the treatments, which occurs when the control is a waiting list or self-help. See the statistical literature for analysis methods^18–21^ and sample size calculations.^22–25^

In the *crossed design*, therapists are crossed by treatment, so that each therapist delivers multiple treatments, which makes it a more efficient design than the nested design.^15^ Another advantage is that it allows for the estimation of the variability of the treatment effect across therapists. The crossed design is in particular feasible in pharmaceutical trials where the new medication is administered to patients using injections or tablets that differ from the placebo only by the amount of active substance. In the ideal case, double blinding is used so that neither the patient nor the health professional knows which treatment the patient receives. Double blinding may eliminate bias due to preferences or expectations with respect to the effect of medication. A parallel can be drawn between a crossed design and a multisite trial.^15,26,27^

This overview of designs is not exhaustive. There are trials in the field of mental health that used designs that are a combination of a nested and crossed design, where one type of health professional delivers just one treatment while another type of health professional delivers multiple treatments.^28–32^ Let us use a trial on treatment of social phobia^29^ as an illustrative example. Cognitive therapy was delivered by clinical psychologists, whereas medication and placebo were delivered by psychiatrists. Even if psychologists were licensed to deliver fluoxetine and placebo, it would not be recommendable to let psychologists actually deliver each treatment because it would be difficult for psychologists to not let patients in the fluoxetine or placebo group benefit from cognitive therapy. However, it could be very well feasible to let psychiatrists deliver both fluoxetine and placebo, especially when double blinding is used. A nested design rather than a crossed design is less efficient.

**Figure 1.**
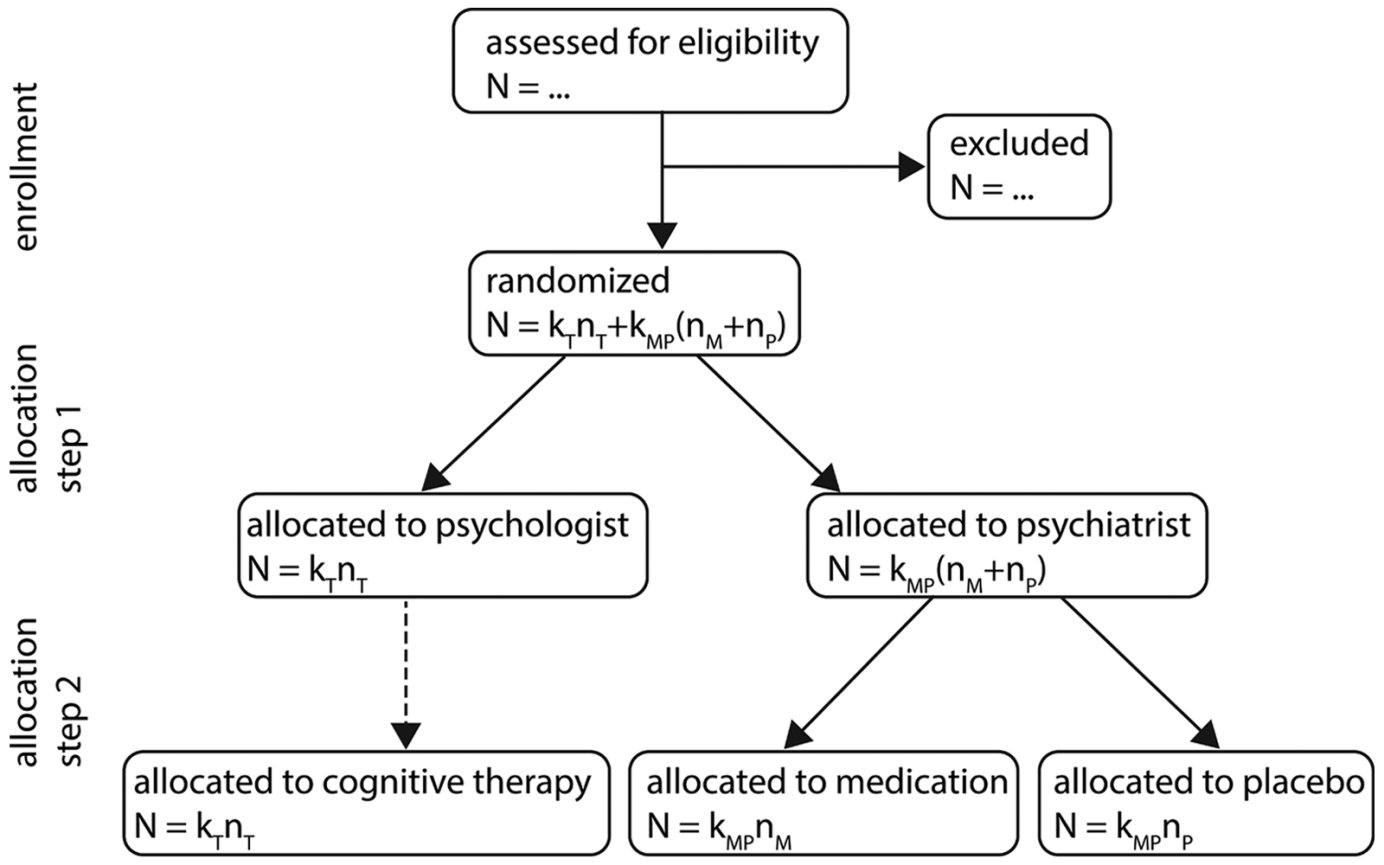
Flow diagram of the multi-tiered experimental design. It is assumed all patients receive allocated treatment. Follow-up and data analysis are not included in this graph. Sample size notation: $k_{T}$ is the number of psychologists, $k_{\mathrm{MP}}$ is the number of psychiatrists, $n_{T}$ is the number of patients per psychologist, $n_{M}$ is the number of patients on medication per psychiatrist and $n_{P}$ is the number of patients on placebo per psychiatrist.

The flow diagram in Figure 1 shows the design is a multi-tiered experimental design since randomization is done in two steps.^33^ First, all eligible patients are randomized to a psychologist or psychiatrist. Second, all patients who were randomized to a psychiatrist are randomized to medication or placebo. Those who were randomized to a psychologist receive cognitive therapy and in fact no randomization in the second step is done for these patients, as is indicated by a dashed arrow.

In this design, the risk of contamination of patients in the medication and placebo groups by those in the cognitive therapy group is minimized, while the efficiency of the comparison of medication and placebo is maximized. It may be considered an interesting alternative to a nested design and has indeed been used in the field of mental health. However, it is also very relevant for other fields where therapy is provided by one type of health professional and is compared to medication and placebo that are provided by another type. Examples are trials to treat excessive alcohol use, binge eating or hypertension. So, even though the remainder of this article uses an example and terminology from mental health, it is also very relevant for practitioners in other fields.

To my knowledge, there are no papers on power and sample size issues for this type of design. A relevant question in the design phase is how many psychologists, how many psychiatrists and how many patients per psychologists and per psychiatrist are required. It is obvious treatment effects are estimated more efficiently when these sample sizes increase, but in practice, they cannot increase without bounds. As an example, the total number of patients may be limited when treatments for a rare disease are compared. It is therefore needed to study which combination of sample sizes results in adequate power. It is important the nesting of patients within health professionals is taken into account while calculating the sample size as ignoring this nested data structure may result in inadequately powered designs. The aim of this article is to provide methodology to calculate the required sample size in a correct way. As such, it helps researchers to plan their trials such that sufficient power is guaranteed and the costs (or total sample size) are minimized.

## Mixed-effects model and statistical power

As outcome scores of patients within the same health professional are dependent, the mixed-effects model should be used for analyzing the data.^34–37^ In addition to that, the variances between and within health professionals may vary across the two types of health professional and three treatment conditions.^2,3,22^ The following mixed-effects model for patient *i* treated by the *j*th health professional takes dependency and heterogeneity into account

(1) $$\begin{array}{c} y_{\mathrm{ij}}=(\mu_{T}+u_{0j}+e_{0\mathrm{ij}})d_{\mathrm{Tj}}+(\mu_{P}+{\tilde{u}}_{0j}+{\tilde{e}}_{0\mathrm{ij}})d_{\mathrm{Pij}}\\ +(\mu_{M}+{\tilde{u}}_{0j}+{\tilde{u}}_{1j}+{\tilde{e}}_{0\mathrm{ij}}+{\tilde{e}}_{1\mathrm{ij}})d_{\mathrm{Mij}} \end{array}$$

Here, $y_{\mathrm{ij}}$ is a quantitative outcome variable and $\mu_{T}$, $\mu_{M}$ and $\mu_{P}$ are the expected mean scores for cognitive therapy, medication and placebo, respectively. The subscripts refer to the types of treatment: *T* for cognitive therapy, *M* for medication and *P* for placebo. The dummy variables $d_{\mathrm{Tj}}$, $d_{\mathrm{Mij}}$ and $d_{\mathrm{Pij}}$ take on the value 1 for a patient within that treatment and the value 0 otherwise. Dummy $d_{\mathrm{Tj}}$ has subscript *j* but not *i* since it varies between health professionals but not within.

These dummies are also used to indicate which random effects are associated with each treatment. The random effects $u_{0j}$ and $e_{0\mathrm{ij}}$ are the between- and within-psychologist effect for cognitive therapy, and ${\tilde{u}}_{0j}$ and ${\tilde{e}}_{0\mathrm{ij}}$ are the between- and within-psychiatrist effect for placebo. The additional random effect ${\tilde{u}}_{1j}$ for the medication group allows the effect of medication versus placebo to vary across psychiatrists. Furthermore, the random effect ${\tilde{e}}_{1\mathrm{ij}}$ is included to allow for heterogeneity across treatments within psychiatrists. The random effects are assumed to follow normal distributions: $e_{0\mathrm{ij}}\sim N(0,\sigma_{0}^{2})$, $u_{0j}\sim N(0,\tau_{0}^{2})$, ${\tilde{e}}_{0\mathrm{ij}}\sim N(0,{\tilde{\sigma }}_{0}^{2})$, ${\tilde{e}}_{1\mathrm{ij}}\sim N(0,{\tilde{\sigma }}_{1}^{2})$, ${\tilde{u}}_{0j}\sim N(0,{\tilde{\tau }}_{0}^{2})$ and ${\tilde{u}}_{1j}\sim N(0,{\tilde{\tau }}_{1}^{2})$. Furthermore, ${\tilde{u}}_{0j}$ and ${\tilde{u}}_{1j}$ are correlated with covariance $\mathrm{cov}({\tilde{u}}_{0j},{\tilde{u}}_{1j})={\tilde{\tau }}_{01}$, ${\tilde{e}}_{0\mathrm{ij}}$ and ${\tilde{e}}_{1\mathrm{ij}}$ are correlated with covariance $\mathrm{cov}({\tilde{e}}_{0\mathrm{ij}},{\tilde{e}}_{1\mathrm{ij}})={\tilde{\sigma }}_{01}$ and all other random effects are independent. A tilde is used to distinguish the random effects and variances for medication and placebo from those for cognitive therapy.

The amount of dependency between outcomes of patients within the same psychologist is quantified by the intraclass correlation coefficient $\rho_{T}=\tau_{0}^{2}/(\tau_{0}^{2}+\sigma_{0}^{2})$. Similarly, in the placebo group, it is ${\tilde{\rho }}_{P}={\tilde{\tau }}_{0}^{2}/({\tilde{\tau }}_{0}^{2}+{\tilde{\sigma }}_{0}^{2})$ and in the medication group, it is ${\tilde{\rho }}_{M}=({\tilde{\tau }}_{0}^{2}+{\tilde{\tau }}_{1}^{2}+2{\tilde{\tau }}_{01})/({\tilde{\sigma }}_{0}^{2}+{\tilde{\sigma }}_{1}^{2}+2{\tilde{\sigma }}_{01}+{\tilde{\tau }}_{0}^{2}+{\tilde{\tau }}_{1}^{2}+2{\tilde{\tau }}_{01})$. These intraclass correlation coefficients do not necessarily have to be equal to each other. In the psychotherapy literature, it is common for preliminary tests to be performed for therapist effects, but this is not recommended since such tests lack power and failure to reject the null hypothesis of no clustering does not rule out the presence of clustering.^4,22^

Fairly simple expressions for the estimators of $\mu_{T}$, $\mu_{M}$ and $\mu_{P}$ and the related covariance matrix can be derived for non-varying number of patients per health professional. The expected means $\mu_{T}$, $\mu_{M}$ and $\mu_{P}$ are simply estimated by taking the average of the outcome scores within each of the three treatments. These averages are calculated across all patients and health professionals, and are denoted as ${\bar{y}}_{T}$, ${\bar{y}}_{M}$ and ${\bar{y}}_{P}$. Double blinding is necessary to get unbiased estimates. The covariance matrix $\mathrm{cov}(\hat{\mu })$ of these estimators is

(2) $$cov\left(\begin{array}{c} {\hat{\mu }}_{T}\\ {\hat{\mu }}_{M}\\ {\hat{\mu }}_{P} \end{array}\right)=\left(\begin{array}{ccc} \frac{\sigma_{0}^{2}+n_{T}\tau_{0}^{2}}{n_{T}k_{T}} & 0 & 0\\ 0 & \frac{{\tilde{\sigma }}_{0}^{2}+{\tilde{\sigma }}_{1}^{2}+2{\tilde{\sigma }}_{01}+n_{M}({\tilde{\tau }}_{0}^{2}+{\tilde{\tau }}_{1}^{2}+2{\tilde{\tau }}_{01})}{n_{M}k_{MP}} & \frac{{\tilde{\tau }}_{0}^{2}+{\tilde{\tau }}_{01}}{k_{MP}}\\ 0 & \frac{{\tilde{\tau }}_{0}^{2}+{\tilde{\tau }}_{01}}{k_{MP}} & \frac{{\tilde{\sigma }}_{0}^{2}+n_{P}{\tilde{\tau }}_{0}^{2}}{n_{P}k_{MP}} \end{array}\right)$$

and an estimate is obtained when the (co)variance components are replaced by their estimates. The entries in equation (2) depend on the sample sizes at the level of the health professional and patient. The numbers of psychologists and psychiatrists are indicated as $k_{T}$ and $k_{\mathrm{MP}}$, respectively. Each psychologist delivers cognitive therapy to $n_{T}$ patients. Each psychiatrist delivers medication to $n_{M}$ patients and placebo to $n_{P}$ patients; hence, the total number of patients per psychiatrist is $n_{\mathrm{MP}}=n_{M}+n_{P}$. The number of psychologists does not necessarily have be equal to the number of psychiatrists, and the number of patients per psychologist does not have to be equal to the total number of patients per psychiatrist. Within each psychiatrist, the number of patients who receive medication can differ from the number of patients on placebo.

The covariance matrix in equation (2) shows that the estimated mean score for cognitive therapy is independent of the mean estimates in the other two treatments because they are delivered by different health professionals. However, the estimated means for medication and placebo are correlated since both treatments are available within each psychiatrist. The precision of the estimated mean for cognitive therapy depends on the number of psychologists and the number of patients per psychologist, but not on the sample sizes in the other two treatments. The precision of the estimated mean for medication depends on the number of psychiatrists and the number of patients per psychiatrist who receive medication, but not on the number of placebo patients per psychiatrist or the sample sizes in the cognitive therapy group. Similarly, the precision of the mean estimate for placebo is only determined by the number of psychiatrists and the number of placebo patients per psychiatrist.

The trial contains three conditions, hence three pairwise comparisons can be made. The effect of medication versus placebo is estimated by the difference in their average outcomes, ${\hat{\mu }}_{M}-{\hat{\mu }}_{P}={\bar{y}}_{M}-{\bar{y}}_{P}$, and the variance of this estimator is

(3) $$\mathrm{var}({\hat{\mu }}_{M}-{\hat{\mu }}_{P})=\frac{{\tilde{\sigma }}_{0}^{2}+{\tilde{\sigma }}_{1}^{2}+2{\tilde{\sigma }}_{01}}{k_{\mathrm{MP}}n_{M}}+\frac{{\tilde{\sigma }}_{0}^{2}}{k_{\mathrm{MP}}n_{P}}+\frac{{\tilde{\tau }}_{1}^{2}}{k_{\mathrm{MP}}}$$

The significance of the difference in means is tested with the test statistic $z=({\hat{\mu }}_{M}-{\hat{\mu }}_{P})/\sqrt{\mathrm{var}({\hat{\mu }}_{M}-{\hat{\mu }}_{P})}$. Under the null hypothesis of no treatment effect, $H_{0}:\mu_{M}=\mu_{P}$, it follows a standard normal distribution, provided the numbers of psychologists and psychiatrists are sufficiently large. For a two-sided alternative hypothesis $H_{a}:\mu_{M}\neq \mu_{P}$ and type I error rate α, the power level 1−β follows from

(4) $$\frac{\mu_{M}-\mu_{P}}{\sqrt{\mathrm{var}({\hat{\mu }}_{M}-{\hat{\mu }}_{P})}}=z_{1-\alpha /2}+z_{1-\beta }$$

where $z_{1-\alpha /2}$ and $z_{1-\beta }$ are the 100(1−α/2) and 100(1−β) percent standard normal deviates. Here, $\mu_{M}-\mu_{P}$ is the population value of the difference in mean outcomes; its value is often unknown in the design phase of a trial and a prior estimate should be provided on the basis of expert knowledge or findings in the literature. Alternatively, it may be replaced by the minimal relevant effect size.

Similar relations between power and effect size can be formulated for the other two pairwise comparisons by making the appropriate changes in equation (4). For the comparison between cognitive therapy and placebo

(5) $$\mathrm{var}({\hat{\mu }}_{T}-{\hat{\mu }}_{P})=\frac{\sigma_{0}^{2}+n_{T}\tau_{0}^{2}}{n_{T}k_{T}}+\frac{{\tilde{\sigma }}_{0}^{2}+n_{P}{\tilde{\tau }}_{0}^{2}}{n_{P}k_{\mathrm{MP}}}$$

For the comparison of cognitive therapy and medication

(6) $$\begin{array}{c} \mathrm{var}({\hat{\mu }}_{T}-{\hat{\mu }}_{M})=\\ \frac{\sigma_{0}^{2}+n_{T}\tau_{0}^{2}}{n_{T}k_{T}}+\frac{{\tilde{\sigma }}_{0}^{2}+{\tilde{\sigma }}_{1}^{2}+2{\tilde{\sigma }}_{01}+n_{M}({\tilde{\tau }}_{0}^{2}+{\tilde{\tau }}_{1}^{2}+2{\tilde{\tau }}_{01})}{n_{M}k_{\mathrm{MP}}} \end{array}$$

### Comparison to a nested design

As both medication and placebo are available within each psychiatrist the variance in equation (3) does not depend on the between-psychiatrist variance component ${\tilde{\tau }}_{0}^{2}$ and covariance component ${\tilde{\tau }}_{01}$. Had a nested design been used, the variance would have been

(7) $$\begin{array}{c} \mathrm{var}({\hat{\mu }}_{M}-{\hat{\mu }}_{P})=\\ \frac{{\tilde{\sigma }}_{0}^{2}+{\tilde{\sigma }}_{1}^{2}+2{\tilde{\sigma }}_{01}+n_{M}^{*}({\tilde{\tau }}_{0}^{2}+{\tilde{\tau }}_{1}^{2}+2{\tilde{\tau }}_{01})}{k_{M}^{*}n_{M}^{*}}+\frac{{\tilde{\sigma }}_{0}^{2}+n_{P}^{*}{\tilde{\tau }}_{0}^{2}}{k_{P}^{*}n_{P}^{*}} \end{array}$$

with $k_{M}^{*}$ and $k_{P}^{*}$ the number of psychiatrists in the medication and placebo groups, respectively, and $n_{M}^{*}$ and $n_{P}^{*}$ the number of patients per psychiatrist in these groups. The relative efficiency is the variance of the crossed design (equation (3)) divided by the variance of the nested design (equation (7)); its value depends on the sample sizes and (co)variance components. A value equal to 1 implies that the nested and crossed design are equally efficient; values below 1 imply that the crossed design outperforms the nested design.

A specific case is a balanced design: $n_{M}=n_{P}=\frac{1}{2}n_{M}^{*}=\frac{1}{2}n_{P}^{*}$ and $k_{M}^{*}=k_{P}^{*}=\frac{1}{2}k_{\mathrm{MP}}$, for which

(8) $$\mathrm{RE}=\frac{\left({\tilde{\sigma }}_{0}^{2}+{\tilde{\sigma }}_{1}^{2}+2{\tilde{\sigma }}_{01})+{\tilde{\sigma }}_{0}^{2}+n_{M}^{*}({\tilde{\tau }}_{1}^{2}/2\right)}{\left({\tilde{\sigma }}_{0}^{2}+{\tilde{\sigma }}_{1}^{2}+2{\tilde{\sigma }}_{01})+{\tilde{\sigma }}_{0}^{2}+n_{M}^{*}(2{\tilde{\tau }}_{0}^{2}+{\tilde{\tau }}_{1}^{2}+2{\tilde{\tau }}_{01}\right)}$$

As this value is always <1, the crossed design outperforms the nested design.

## Finding the cost-efficient design

### Design space

The power of the test for a pairwise comparison depends on the design ξ, which is the combination of sample sizes $\xi =(n_{T},n_{M},n_{P},k_{T},k_{\mathrm{MP}})$. In practice, these sample sizes are often limited by some constraints. For instance, the number of psychologists and psychiatrists who are available for the trial may be limited to some maximum values. The design space is determined by all combinations ξ of sample sizes that do not exceed their maximum values. Different designs may result in the same power level, hence, it is reasonable to take costs into account while selecting the cost-efficient design.

### Costs of a trial

The costs of a trial depend on the costs for training and supervising health professionals and for treating and measuring patients. The costs for training and supervising one psychologist to deliver cognitive therapy are denoted as $c_{2T}$, and these costs are independent of the number of patients treated per psychologist. The costs for treating and measuring one patient in the cognitive therapy group are denoted as $c_{1T}$. The numbers in the subscripts of these costs refer to the level in the hierarchical data structure: the patient level is the first level and the health professional level is the second. The letter in the subscripts refers to the type of treatment. In a similar manner, the costs per psychiatrist are denoted as $c_{2\mathrm{MP}}$, the costs per patient who receives medication are denoted as $c_{1M}$ and the costs per patient who receives placebo are denoted as $c_{1P}$. The costs are given by

(9) $$C=(c_{2T}+c_{1T}n_{T})k_{T}+(c_{2\mathrm{MP}}+c_{1M}n_{M}+c_{1P}n_{P})k_{\mathrm{MP}}$$

A special case of equation (9) is achieved when $c_{2T}=c_{2\mathrm{MP}}=0$, when $c_{1T}=c_{1M}=c_{1P}=1$ and *C* is replaced by *N*

(10) $$N=n_{T}k_{T}+(n_{M}+n_{P})k_{\mathrm{MP}}$$

In this case, the total number of patients is used to select the design. This is relevant when the trial compares treatments for a rare disorder and where the number of patients is limited but costs are less relevant.

### Finding the cost-efficient design

The cost-efficient design is found by evaluating all possible combinations of sample sizes $k_{T}$, $k_{\mathrm{MP}}$, $n_{T}$, $n_{M}$ and $n_{P}$ that do not exceed their maximum values. For each combination, the power levels for the three pairwise comparisons are calculated, as well as the costs (or total number of patients). Out of those designs that have sufficient power for each of the three pairwise comparisons, the design is selected that has smallest costs (or smallest total sample size). This is the cost-efficient design and it can be found by using an Internet application at https://utrecht-university.shinyapps.io/cost-efficient-designs/. The R code underlying this application is available from the author.

### Conditional designs

In some studies, one or more sample sizes may be fixed to a constant. The number of patients per health professional may be fixed based on the professionals’ work schedules. The number of health professionals may be fixed due to contracts that were made while planning the trial. Such designs are referred to as conditional optimal designs^38^ and they are in general more expensive than the cost-efficient design. They can be found by using the same Internet application.

## Example: placebo-controlled comparison of treatments for social phobia

A total of 60 patients with social phobia were randomly assigned to cognitive therapy, fluoxetine plus self-exposure, or placebo plus self-exposure.^29^ Each treatment was delivered to 20 patients, and allocation to fluoxetine or placebo was double-blind. Cognitive therapy was delivered by four experienced clinical psychologists, so the average number of patients per psychologists was five. Fluoxetine and the placebo were delivered by four psychiatrists, so on average 10 patients were treated by each psychiatrist.

Patients had up to 16 weekly treatment sessions; measurements on 10 quantitative outcome variables were taken at baseline, halfway treatment and posttest. Analyses were intent to treat. One-way analyses of variance were performed to identify any differences between treatment groups before the start of treatment. One-way analyses of covariance, with pretreatment scores as covariate, were performed at the next two measurements. This article did not mention any strategies to deal with the nesting of patients within psychologists and psychiatrists.

The Beck Anxiety Inventory as measured at posttest will be used to illustrate the design methodology. The average outcome in the cognitive therapy group was ${\bar{y}}_{T}=5.50$ (SD = 5.93); in the fluoxetine group, it was ${\bar{y}}_{M}=7.95$ (SD = 7.20); and in the placebo group, it was ${\bar{y}}_{P}=9.50$ (SD = 7.32). No significant between-treatment differences on the mean scores were found: F(2,56)=1.6(p=0.21), which corresponds to a medium effect size ($\eta^{2}=0.054$). Cohen’s *d* for the comparison of cognitive therapy and fluoxetine was 0.37; for the comparison of cognitive therapy and placebo, it was 0.60; and for the comparison of fluoxetine and placebo, it was 0.21. These effects are small to medium in size.^39^ Overall, it is not surprising no significant effects were detected for this outcome variable with a total sample size of just 60.

Assume this study is to be replicated in a larger study such that power levels of at least 80% are achieved for all pairwise comparisons while costs are minimized. The estimates for the means and standard deviations as given above are used in finding the design. Values of the intraclass correlations coefficients were not provided. Baldwin et al.^40^ investigated intraclass correlation coefficients for a variety of outcomes in psychotherapy trials. The mean estimate for the Beck Depression Inventory was $\rho_{T}=0.049$ and the same value will be used here for the Beck Anxiety Inventory. For the other two treatments, an intraclass correlation coefficient that is twice as high is used: ${\tilde{\rho }}_{P}={\tilde{\rho }}_{M}=0.1$. This implies a lower correlation between patients within a psychologist than within a psychiatrist, which may be a result of standardization of cognitive therapy. Furthermore, the variance of the within-psychiatrist effect of medication versus placebo is set at ${\tilde{\tau }}_{1}^{2}=0.05$, which implies that 95% of the treatment differences are within the predictive interval ${\bar{y}}_{P}-{\bar{y}}_{M}\pm z_{0.975}*{\tilde{\tau }}_{1}=[1.11,1.99]$.

The following costs are used: $c_{2T}=1000$, $c_{2\mathrm{MP}}=250$, $c_{1T}=200$, $c_{1M}=200$ and $c_{1P}=20$. It is reasonable to assume that costs to train a psychologist to deliver a new type of cognitive therapy are higher than costs to train a psychiatrist to deliver a new type of medication or placebo. Furthermore, it is reasonable to assume that patient-level costs for cognitive therapy and medication are higher than costs for placebo. In this example, the patient-level costs for cognitive therapy and medication are equal, but this is not always the case.

Table 1 lists the three scenarios that are used in this example. In the first scenario, all sample sizes have upper limits, where the maximum number of patients per psychologist is less than the maximum total number of patients per psychiatrist. This reflects the fact that cognitive therapy is more time-consuming to deliver. In the second scenario, the number of health professionals is fixed to a constant, while in the third scenario, the number of patients per health professional is fixed. Hence, in the latter two scenarios, we seek conditional optimal designs.

**Table 1.** Description of three scenarios in the example on social phobia.

Scenario	Restrictions			
1	$k_{T}\leq 30$	$k_{\mathrm{MP}}\leq 30$	$n_{T}\leq 20$	$n_{\mathrm{MP}}\leq 30$
2	$k_{T}=25$	$k_{\mathrm{MP}}=25$	$n_{T}\leq 20$	$n_{\mathrm{MP}}\leq 30$
3	$k_{T}\leq 30$	$k_{\mathrm{MP}}\leq 30$	$n_{T}=15$	$n_{\mathrm{MP}}=25$

The cost-efficient designs for these three scenarios are given in Table 2, along with their total sample size, costs and power levels for the three pairwise comparisons. For scenarios 1 and 3, the number of psychologists is lower than the number of psychiatrists, which is not surprising given the higher costs to train and supervise a psychologist. For a similar reason, the number of patients in the placebo group is higher than the number of patients in the medication group. In all three scenarios, a psychiatrist treats more patients than a psychologist.

**Table 2.** Cost-efficient designs for the three scenarios in the example on social phobia.

Scenario	$k_{T}$	$k_{\mathrm{MP}}$	$n_{T}$	$n_{M}$	$n_{P}$	Total *N*	Costs	$\mathrm{powe}r_{\mathrm{TM}}$	$\mathrm{powe}r_{\mathrm{TP}}$	$\mathrm{powe}r_{\mathrm{MP}}$
1	13	30	11	7	20	953	103100	0.80	1.00	0.80
2	25	25	5	9	20	850	111250	0.81	1.00	0.80
3	11	29	15	8	17	890	107510	0.81	1.00	0.81

The design for scenario 1 has the lowest costs but the highest total sample size. The costs for the other two scenarios are higher than those for scenario 1 because these are conditional designs. However, the difference in costs is only minor while the conditional designs include fewer patients. For each scenario, the comparison of cognitive therapy versus placebo has highest power, and the power levels for the other two comparisons are about the desired value 0.8. Furthermore, for each scenario, the total sample size is much higher than the total of 60 patients in the original study.

## Conclusion and discussion

This article provides the methodology to calculate optimal sample sizes in trials with one or two treatments per health professional. Optimal sample sizes are calculated such that sufficient power is achieved at minimal costs or minimal total sample size. The optimal design does not necessarily assign equal number of patients to each treatment condition, neither is the number of psychologists necessarily equal to the number of psychiatrists. In the illustrative example, the optimal sample sizes reflect the costs for the different treatment conditions and for both types of health professionals.

The sample sizes are calculated based on the mixed model (1) that explicitly takes into account the nesting of patients within health professionals. This model should also be used for analyzing the data once the trial has been executed. Ignoring the hierarchical nature of the data may result in underestimates of the standard errors of treatment effect sizes and hence inflated type I error rates.^41^ The specific feature of model (1) is that it needs treatment indicators in its random part to account for heterogeneity.

The mixed model allows the effect of medication versus placebo to vary across psychiatrists. Given that the design is double-blind, one may argue if such variation is plausible in all practical settings. Psychiatrists may vary with respect to the amount of emphasis they put on the importance of treatment adherence. As a result, patients’ treatment compliance, and hence treatment effect estimates in an intention to treat analysis, may vary across psychiatrists. Psychiatrists may also vary with respect to the amount of attention they pay to their patients and how well they are able to reassure them. Such attention and reassurance may be of importance in trials that treat some psychological disorder, such as anxiety. If such attention and reassurance strengthen the effect of medication, then between-psychiatrist variability in attention and reassurance may result in treatment effects that vary across psychiatrists, even in the case of double blinding. However, when the effect of treatment is physiological in nature, then the effect of treatment may probably not vary. As an example, one can think of the effect of growth hormone versus placebo on final body height of adolescents with growth retardation. If there are plausible reasons to assume treatment variation is absent, then the model and Internet application can still be used by setting $\tau_{1}^{2}=0$. Otherwise, it is suggested to take the possibility of treatment effect variation into account while calculating sample sizes to avoid underpowered studies and while analyzing the data to avoid inflated type I error rates.

The flow diagram in Figure 1 assumes random assignment in both steps. The order of these steps may also be reversed such that patients are first randomized to treatments and subsequently randomized to health professionals. Random allocation of patients to health professionals is important if confounding of therapist variation by patient characteristics is to be avoided. Random assignment is not always possible, for instance, when patients are recruited in real time and allocated to the next available therapist, or when it is practical or desirable to maintain pre-existing therapist–patient allocations. Non-random assignment would not change the data structure or the model, but it may affect the standard errors of intervention effect estimates. See also the section on internal validity in Walwyn and Roberts.^5^

It should be noted that each psychologist treats $n_{T}$ patients and each psychiatrist treats $n_{\mathrm{MP}}$ patients. The effect of varying number of patients per health professional may be studied in future research. Thus far, it has been shown that sampling 11% more clusters often suffices when cluster sizes vary in cluster randomized trials or individually randomized trials with partial clustering.^42,43^

The optimal design may include a very low number of health professionals. In such cases, the variance components at the level of the health professional may be estimated with bias, which in its turn may have an effect on significance of treatment effects. In such cases, one may consider alternatives to the multilevel model, such as the fixed-effects model.^44^

The design is restricted to the case where there is one health professional delivering care to each patient but this is not always the case. There are situations in which patients receive therapy that consists of multiple sessions delivered by therapists of the same type, creating a multiple membership structure.^35,45^ Another example is an intervention that consists of different components, which are each delivered by therapists of different types, so patients are crossed by therapist.^45^ Further levels are introduced when several therapists deliver a group treatment or when patients are nested within pre-existing groups, such as general practices or clinics, that are crossed by therapists.^46^

To calculate the optimal sample sizes, the values of the total variances and intraclass correlations in each treatment need to be known a priori. These values are often unknown in the design phase of a trial and have to be replaced by an educated guess from experts’ opinions or findings in the literature. For cluster randomized trials, a large amount of papers that list estimates of intraclass correlation coefficients have been published over the past 20 years.^47^ Such papers should also be published for the design that is considered in this article.
